# The L-Cell in Nutritional Sensing and the Regulation of Appetite

**DOI:** 10.3389/fnut.2015.00023

**Published:** 2015-07-20

**Authors:** Eleanor Spreckley, Kevin Graeme Murphy

**Affiliations:** ^1^Section of Investigative Medicine, Department of Medicine, Imperial College London, Hammersmith Hospital, London, UK

**Keywords:** enteroendocrine, appetite, glucagon-like peptide-1, peptide YY, macronutrient

## Abstract

The gastrointestinal (GI) tract senses the ingestion of food and responds by signaling to the brain to promote satiation and satiety. Representing an important part of the gut–brain axis, enteroendocrine L-cells secrete the anorectic peptide hormones glucagon-like peptide-1 (GLP-1) and peptide YY (PYY) in response to the ingestion of food. The release of GLP-1 has multiple effects, including the secretion of insulin from pancreatic β-cells, decreased gastric emptying, and increased satiation. PYY also slows GI motility and reduces food intake. At least part of the gut–brain response seems to be due to direct sensing of macronutrients by L-cells, by mechanisms including specific nutrient-sensing receptors. Such receptors may represent possible pathways to target to decrease appetite and increase energy expenditure. Designing drugs or functional foods to exploit the machinery of these nutrient-sensing mechanisms may offer a potential approach for agents to treat obesity and metabolic disease.

## Introduction

The gastrointestinal (GI) tract represents the largest endocrine organ in the human body. Enteroendocrine cells (EECs) are located throughout the GI tract, constituting <1% of the cell population in the intestinal epithelium, but playing critical physiological roles and representing an important component of the gut–brain axis (1). At least 15 types of EEC have been described, capable of secreting over 20 peptide hormones that influence processes including gut motility, gastric acid secretion, and energy intake. It was previously thought that EECs could be separated into discrete classes of cells with specific secretory profiles. Examples of previously characterized cell families include gastrin-secreting G-cells, cholecystokinin (CCK)-secreting I-cells, glucagon-like peptide (GLP-1), and peptide YY (PYY)-secreting L-cells, among others (2). However, recent work has suggested that EEC families may be less well defined, with EECs existing as a wide range of cell types that secrete various combinations of different peptides (3).

**Figure 1:**
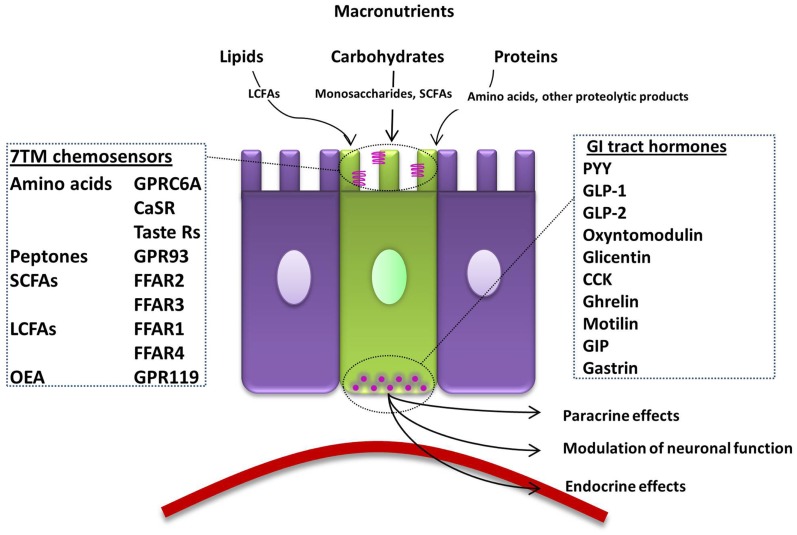
**Summary of the macronutrient sensing receptors expressed in enteroendocrine cells of the gut and the hormones they release**. An open-type enteroendocrine cell possesses microvilli extending into the gut lumen, coming into direct contact with macronutrients. Food components are sensed by various GPCRs and transporters located on the apical border. GI tract hormones are released into the circulation, acting via paracrine, endocrine, and neural pathways to modulate food intake. LCFA; long-chain fatty acid, 7TM; 7-transmembrane, CaSR; calcium-sensing receptor, GPRC6A; G-protein coupled receptor family C group 6 subtype A, GPR93; G-protein coupled receptor 93, SCFA; short-chain fatty acid, FFAR; free-fatty-acid receptor, OEA; oleoylethanolamide, GPR119; G-protein coupled receptor 119, GI; gastrointestinal, PYY; peptide YY, GLP; glucagon-like peptide, CCK; cholecystokinin, GIP; gastric inhibitory peptide.

Nervous and endocrine signaling between the gut and the brain allows the modulation of GI functions to increase the efficiency of digestion, and the communication of energy and nutritional requirements to the brain to influence appetite. One key function of EECs is to sense luminal contents, which will then modulate their release of hormones that regulate food intake. To achieve this, open-type cells often have a distinct cone-shaped morphology with one extremity adjacent to the basal lamina and the other possessing microvilli on apical processes (Figure 1). Microvilli are thus in immediate contact with the luminal contents, sensing of which can lead to the release of hormones from secretory granules directly into the nearby blood vessels (4). G-protein coupled receptors (GPCRs) represent over one-third of therapeutic drug targets, and a number detect dietary components. When the products of food breakdown move through the GI tract, specific macronutrients stimulate the chemosensors of a variety of GPCRs. This leads to the modulation of gut hormone release, which will influence neuronal signaling in appetite centers in the brain to mediate the appropriate feeding behavior, by, for example, the termination of hunger and the induction of satiety (5). In contrast, closed-type EECs do not come into contact with luminal nutrients, and instead react to neural or circulating signals, though they also play a role in the regulation of food intake. In addition, the hormones released from EECs can have paracrine effects on nearby cells, including neurones. Recent evidence also suggests that EECs interact directly with neurones via synapse-like structures named neuropods (6, 7).

The human body stores excess energy intake as adipose tissue. The present obesity pandemic is a major global health issue, which has arisen due to the abundance of highly palatable, calorie-dense food combined with reduced levels of physical activity. The pharmacological agents for weight loss currently available are only modestly effective. Having previously been advocated for use in the morbidly obese, bariatric surgery is now recommended for obese patients with type II diabetes in UK (8). However, the number of patients that now qualify suggests that this is an impractical approach to dealing with obesity. Targeting gut hormone receptors to decrease appetite and increase energy expenditure is a major area of interest for the management of body weight (9–11), and the GLP-1 receptor agonist Liraglutide (Saxenda) has recently been approved by the U.S. Food and Drug Administration as a treatment for obesity (12). However, the formulation of foodstuffs, which contain targeted nutraceuticals to exploit the various nutrient-sensing systems, present on EECs represents another possible approach to the treatment of obesity, which might avoid the problems of administration, nausea, and tachyphylaxis that have been associated with gut hormone administration (Table 1) (13–15).

**Table 1:** **A summary of potential targets for the treatment of obesity and their mechanisms**.

Potential targets	Mechanism	Reference
Oxyntomodulin	GLP-1 and glucagon receptor agonism	(10, 16)
Peptide YY	Y2R agonism modulates central anorectic pathways and influences ileal brake	(17, 18)
Dietary supplementation with glutamine and l-arginine	Ingested glutamine and l-arginine potentiate the release of GLP-1 and PYY, via activation of AMPK and mTOR	(19–22)
Calcium-sensing receptor	Activation by specific l-amino acids stimulates the secretion of GLP-1 and PYY	(23)
G-protein coupled receptor 93	Protein hydrolyzates stimulate the release of CCK	(24)
G-protein coupled receptor, class C, group 6, subtype A	Activation by specific l-amino acids stimulates the secretion of GLP-1	(25, 26)
Sodium-glucose transporter 1	Transport of ingested glucose into enterocytes stimulates the secretion of GLP-1	(27)
Free fatty acid receptor 2 and 3	Activation by short-chain fatty acids may stimulate the secretion of GLP-1 and PYY and inhibit gastrointestinal motility	(28–30)
Free fatty acid receptor 1 and 4	Activation by medium and long-chain fatty acids stimulates the secretion of GLP-1	(31–33)

## The Hormonal Products of L-Cells

Gastric distension and the release of upper-intestinal tract hormones, such as CCK from I-cells, trigger short-term satiation processes in the upper GI tract (4). However, longer-term satiety is likely driven by other mechanisms, which may include the direct sensing of nutrients leading to the release of anorectic gut hormones from enteroendocrine L-cells. Mature L-cells are commonly defined as EECs that express the preproglucagon gene. Posttranslational processing of preproglucagon is tissue-specific, and hence, yields different hormonal products in the pancreas and intestine. L-cells, which have traditionally been described as having a distinct cone-shaped morphology, secrete the products of cleavage by prohormone convertase 1; GLP-1, glucagon-like peptide 2 (GLP-2), glicentin, and oxyntomodulin (34–36). EECs have been suggested to express an overlapping spectrum of hormones dependent on spatial distribution and exposure to nutrients (3, 37). Immunostaining and fluorescence-activated cell sorted analysis (FACS) have revealed that L-cells co-secrete distinct peptides depending on their location. L-cells in the upper small intestine demonstrate co-localization with gastric inhibitory peptide (GIP), and thus, bear some resemblance to neighboring K-cells, while L-cells in the lower small intestine show high levels of co-localization with PYY and CCK. The GLP-1 and PYY co-expressing L-cells are typically considered to be involved in the regulation of energy homeostasis, in addition to other functions. GLP-1 mediates its effects via the GLP-1 receptor. PYY exists in two major circulating forms: the full-length peptide, PYY_1–36_, and a truncated form, PYY_3–36_. Full-length PYY acts on Y family receptors Y1, Y2, and Y5, whereas PYY_3–36_ is relatively selective for the Y2 receptor (38, 39).

L-cells that co-express GLP-1 and PYY are located along much of the length of the GI epithelium, starting at the proximal jejunum and increasing in density along the small intestine and then the large intestine. Thus, the contact of ingested nutrients with L-cells increases along the GI tract (34, 40). GLP-1 and PYY exhibit a two-phase release profile. The initial rapid rise in GLP-1 may partially represent release from L-cells in the upper small intestine. However, it is thought that most of this first phase response for GLP-1 and that of PYY is mediated via a neural reflex or a circulating factor (41, 42). The arrival of food in the distal gut is thought to drive the second phase of the release of GLP-1 and PYY into the circulation, by activation of specific nutrient receptors and other cellular machinery present on apical cell processes (43, 44).

## Hormonal Products of L-Cells: Peripheral Effects

L-cell-secreted GLP-1 and PYY diffuse into the lamina propria and enter the systemic circulation via the hepatic portal vein. Systemic circulating levels of both hormones rise within 15 min of food ingestion in humans, with levels approximately proportional to the calories ingested. Following a mixed meal, plasma concentrations of GLP-1 and PYY peak at around 40 and 90 min, respectively, then reach a plateau (45, 46). GLP-1 and PYY circulate at basal levels in the fasting state, with concentrations rising rapidly postprandially; an effect that seems to be larger in humans than in rodents (41, 47–50). Following release, both GLP-1 and PYY undergo enzymatic cleavage in the intestinal endothelium and liver by dipeptidyl peptidase IV (DPPIV), which converts GLP-1 to an inactive form, and truncates PYY_1–36_ to its Y2 selective form, PYY_3–36_.

### Glucagon-like peptide-1

**Figure 2:**
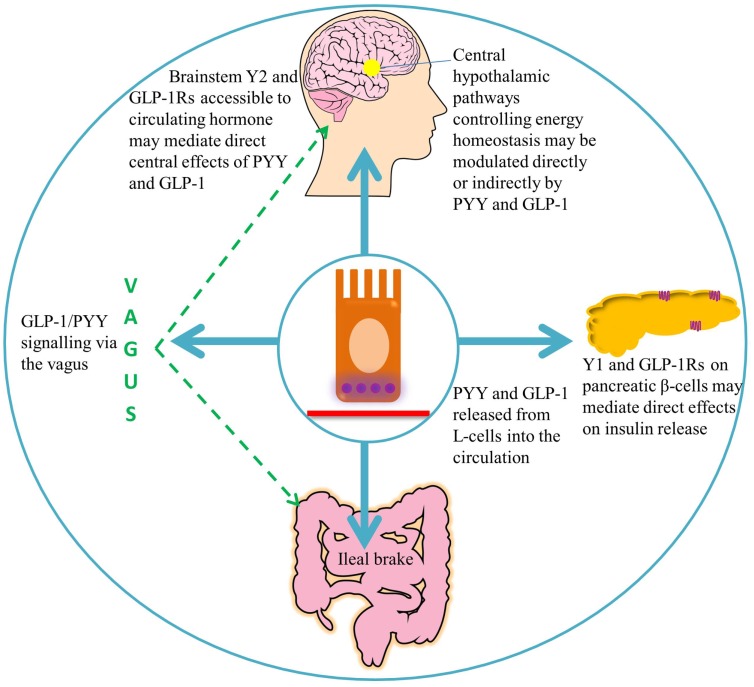
**A summary of the target areas of L-cell secreted PYY and GLP-1**. Following nutrient ingestion, PYY and GLP-1 diffuse into the lamina propria and enter the circulation. GLP-1 binds its receptors on pancreatic β-cells, leading to insulin secretion (51). Full-length PYY binds pancreatic Y1 receptors and inhibits glucose-stimulated insulin secretion, while PYY_3–36_ may exert effects on glucose homeostasis via extra-islet Y2 receptors (56, 57). Circulating hormones are able to access areas of the hindbrain with a leaky blood–brain barrier, such as the area postrema, which communicates with the nucleus of the solitary tract (58). GLP-1 and PYY_3–36_ may signal via the vagus to central hypothalamic nuclei controlling energy homeostasis, where receptors for these hormones are widely expressed (46, 59–61).

The release of GLP-1 has several peripheral consequences, the most notable being its incretin effect. GLP-1 and GIP are reported to bind their respective receptors on β-cells in the pancreas in response to glucose (Figure 2). This leads to an increase in the concentration of intra-cellular calcium, and consequent exocytosis of insulin-containing vesicles (51). Glicentin has a similar but less potent effect, though a glicentin-specific receptor remains to be identified (52, 53). However, there is some controversy surrounding the incretin role of GLP-1 due to the relatively small increase in circulating GLP-1 observed postprandially, and the short lifespan of the peptide (54). Specific knockdown of *Glp1r* in the pancreatic β-cells of mice impairs glucose tolerance in response to hyperglycaemmia, and attenuates insulin secretion in response to exogenous GLP-1. However, a DPPIV inhibitor retained its glucose lowering effects in these mice, suggesting that the GLP-1R on the beta cell is not necessary for these effects, and that perhaps extra-islet receptors are responsible for the incretin effects of GLP-1. However, this may also reflect compensatory action by other DPPIV substrates (55).

**Figure 3:**
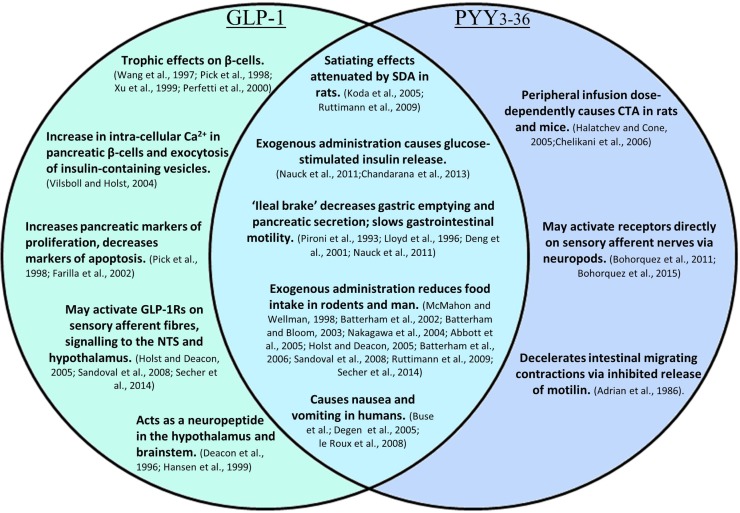
**A summary of the effects of GLP-1, PYY_3–36_, and the combined effects of both**.

In addition to acute effects, GLP-1 exhibits trophic effects on β-cells (62–65); a 2-day infusion of GLP-1 increased markers of proliferation, and decreased markers of apoptosis in the pancreas of Zucker diabetic rats (Figure 3) (66). This is thought to be mediated by the activation of transcription factors that result in enhanced proliferation and differentiation, and inhibition of apoptosis, thereby promoting islet growth (63, 66).

Vagal nervous activity can reduce gastric emptying, thus, slowing nutrient absorption. The slowing of gastric emptying by GLP-1 is subject to rapid tachyphylaxis following chronic exposure, likely at the level of the vagus nerve. In human, subjects given a continuous intravenous infusion of GLP-1, post-prandial concentrations of glucose, glucagon, and insulin progressively increased in subsequent meals. Therefore, it is possible that part of the glycemic control afforded by administration of GLP-1 occurs secondary to delayed gastric emptying (14). The related peptide, GLP-2, is co-secreted with GLP-1, and contributes to optimizing the local environment for nutrient absorption. GLP-2 induces crypt cell proliferation, while also preventing the apoptosis of intestinal cells and increasing GI blood flow (67, 68). Peripheral administration of GLP-2 also increases the expression of cellular transport machinery involved in glucose absorption in the rat jejunum, such as the sodium-glucose transporter 1 (SGLT-1) and glucose transporter 2 (GLUT2) (69, 70).

Originally demonstrated by an infusion of corn oil, administration of carbohydrate, lipid, and protein directly into the ileum of humans also stimulates the “ileal brake,” which results in the secretion of PYY and GLP-1, reduced food intake, and satiation (71–73). The ileal break is a feedback mechanism initiated by the presence of unabsorbed dietary components in the ileum, and acts to slow more proximal GI motility in order to allow efficient digestion and uptake of nutrients. GLP-1 and PYY appear to be important components of this system (Figure 2). Exogenous administration of PYY_3–36_ or GLP-1 decreases gastric emptying and pancreatic secretion (14, 18, 74) and decreases the speed of intestinal transit, an effect, which may partly be due to the inhibited release of motilin by PYY, and a consequent decrease in intestinal migrating contractions (17, 75). However, activation of Y1 receptors by PYY_1–36_ tonically accelerates colonic transit, an effect, which is attenuated by antagonism of this receptor (76), and which is perhaps designed to empty the colon to allow it to deal with the nutrients coming down the small intestine. Binding of PYY_1–36_ to the Y1 inhibitory receptor subtype in the gastric mucosa also regulates the function of gastric endocrine cells, inhibiting vagally stimulated gastric acid secretion (77).

### Peptide YY

In addition to appetite regulation, PYY may regulate pancreatic islet function to regulate glucose homeostasis. Full-length PYY_1–36_ inhibits glucose-stimulated insulin secretion from murine islets, an effect thought to be mediated via neuropeptide Y (NPY) Y1 receptors. In accord with this, *Pyy*-null mice, or islets in which the Y1 receptor is absent, hypersecrete insulin (56, 57). The absence of the Y2 receptor in human and murine pancreatic islets corresponds to the lack of a direct effect of PYY_3–36_ on glucose-stimulated insulin secretion (78). However, the peripheral administration of exogenous PYY_3–36_ or Y2R agonist, administered either in the fed state or in combination with glucose, improved glucose tolerance, and stimulated insulin secretion in mice. This effect was abolished by the administration of a peripheral Y2R antagonist, implying that PYY_3–36_ exerts its effects on glucose homeostasis via extra-islet Y2 receptors in a nutrient-dependent manner (57). Endogenous PYY_3–36_ may act via the Y2R to increase hepato-portal GLP-1, as the effect was attenuated by administration of the GLP-1R antagonist, Exendin9-39 (57). However, a combined intravenous infusion of PYY_3–36_ and GLP-1 to overweight subjects increased first phase insulin secretion to the same level as GLP-1 alone; thus, the addition of PYY_3–36_ had no additive effects at the doses tested in an acute setting (79).

### Oxyntomodulin

Oxyntomodulin, a longer isoform of glucagon, is a dual agonist of the GLP-1 and glucagon receptors, though with a 10- to 100-fold lower affinity than the native ligands (16, 80, 81). Unsurprisingly, the effects of oxyntomodulin combine those of GLP-1 and glucagon. Centrally injected oxyntomodulin reduces food intake to the same magnitude as GLP-1, despite a much lower receptor affinity (16). Exogenous administration reduces food intake in rodents and humans, an effect mediated by the GLP-1 receptor, and also modulates gastric acid and exocrine pancreatic secretion (82–84). Chronic pre-prandial administration of oxyntomodulin drove significant weight loss in overweight and obese human subjects (10). Studies in pair-fed rats suggested that, in addition to decreased energy intake, oxyntomodulin-stimulated weight loss may be the result of increased energy expenditure (84). Subsequent human data confirmed that oxyntomodulin increased activity-related energy expenditure, though not resting energy expenditure, following pre-prandial subcutaneous self-administration in overweight subjects (10). The beneficial actions of oxyntomodulin on food intake, energy expenditure, and body weight place it at the forefront of peptides of interest in the treatment of obesity. Modifying the N-terminus of oxyntomodulin, which confers resistance to enzymatic degradation by DPPIV, has shown significant therapeutic potential in terms of improved glycemic control and appetite suppression (85, 86).

## Hormonal Products of L-Cells: The Gut–Brain Axis

A number of brain regions are involved in the central regulation of energy homeostasis, including the hypothalamus, the cortex, the limbic system, and the brain stem (87). The hypothalamus is divided into distinct nuclei that co-ordinate orexigenic and anorexigenic signals. The arcuate nucleus (ARC) is located proximate to the median eminence, an area with an incomplete blood–brain barrier, and is therefore in contact with circulating factors, including PYY and GLP-1. Within the ARC two distinct sets of neurons have opposing effects on appetite. Activation of pro-opio melanocortin (POMC) neurons inhibits appetite, while activation of NPY/agouti-related peptide (AgRP) neurons stimulates appetite (88–91). These neuronal populations project to intra- and extra-hypothalamic regions to regulate energy homeostasis. The nucleus of the solitary tract (NTS) in the brainstem receives input from the periphery via vagal afferent fibers, transmitting information, such as gastric distension, ingested dietary composition, and water content. Vagal efferent fibers are located in the dorsal motor nucleus, which is located ventral to the NTS in the caudal brainstem (92). These brain regions are critical to the control of energy homeostasis, and are thus the target regions for central pharmacological manipulation, with the hope that drugs acting specifically in these areas will display fewer unwanted side-affects.

### GLP-1 in the gut–brain axis

The rapid breakdown of GLP-1 results in a half-life of approximately 2 min. Concentrations of GLP-1 are therefore highest in the intestinal submucosa, decreasing in the hepatic portal vein and systemic circulation. An estimated 15% of active GLP-1 secreted from the porcine intestine reaches the systemic circulation, before it is also degraded by DPPIV (93). Consequently, there is debate as to whether physiologically relevant levels in men are able to reach central GLP-1 receptors in the hypothalamus and brainstem, where centrally produced GLP-1 is also thought to act as a neuropeptide (94, 95). It has been suggested that peripheral GLP-1 may act via a neural rather than an endocrine route, activating receptors near its site of release before the peptide encounters endothelial DPPIV. Receptors for GLP-1 are located on enteric neurons that exhibit increased action potential firing in primary culture following the application of GLP-1, which may, for example, inhibit local muscle tone as part of the ileal brake (96). Receptors on sensory afferent fibers of the nodose ganglion may also be activated, relaying impulses to central regions important in energy homeostasis, such as the NTS and the hypothalamus (93). Expression of the GLP-1 receptor in neuronal cells of the ganglion has been confirmed by Nakagawa et al., who also demonstrated that intraportal injection of physiological levels of GLP-1 increased afferent signaling of the rat hepatic vagus, providing evidence for vagal chemoreception of peripheral GLP-1 (96, 97). Interestingly, rats that had undergone subdiaphragmatic vagal deafferation were less sensitive to the anorectic effects of intraperitoneally administered GLP-1, whereas the effects of GLP-1 administered into the vena cava and hepatic portal vein were not affected over a wide range of doses. This suggests that while intraperitoneal GLP-1 requires abdominal vagal afferent signaling to exert its anorectic effects, circulating GLP-1 mediates these effects via an alternative mechanism (Figure 3) (98). It has been suggested that exogenous intraperitoneal GLP-1 acts in a similar way to endogenous L-cell-secreted GLP-1, acting in a paracrine fashion, before DPPIV denatures the peptide within the capillary walls (96, 98).

As the satiating effects of intravenous GLP-1 are unaffected by vagotomy, it is possible that GLP-1 administered in this way is acting directly at central GLP-1 receptors (98, 99). Circulating GLP-1 is able to diffuse across the fenestrated capillaries of the circumventricular organs, binding receptors in the subfornical organ and area postrema (AP) (58). The AP is positioned in close proximity to the NTS, to which it communicates nutritional information. The AP also has efferent and afferent connections to the hypothalamus, allowing it to moderate feeding in response to the nutritional demands (58, 100). The GLP-1 receptor is widely expressed throughout the hypothalamus, with the highest expression in the PVN and ARC (60, 61). This coincides with dense connections from the NTS, which also expresses preproglucagon, highlighting the importance of the brainstem–hypothalamic pathway in GLP-1 signaling (60). The PVN is thought to be the primary mediator of brain-derived GLP-1-induced satiation, as direct injection into this nuclei elicits a robust anorectic response (101, 102). GLP-1 may also elicit its effects on food intake via the ARC, with some POMC neurons expressing the GLP-1 receptor (102). Indeed, evidence suggests that peripheral administration of liraglutide, a long-acting GLP-1 analog, acts on ARC POMC neurons to drive weight loss (61). The central expression of preproglucagon is highly conserved between rodents and non-human primates. Mapping of the GLP-1 receptor in the non-human primate brain is consistent with the functional role of GLP-1, with the most abundant expression in areas controlling energy homeostasis, specifically the hypothalamic and brainstem regions mentioned above. Interestingly, a higher level of expression of the GLP-1 receptor was present in the amygdala of the primate brain compared with the rodent brain, indicating a possible species difference in GLP-1 signaling in this region (103).

### PYY in the gut–brain axis

Peptide YY has a half-life of approximately 10 min in humans, though plasma levels remain increased for up to 6 h postprandially due to sustained release (46, 104). The Y5 receptor is expressed throughout the brain, though most densely in the hypothalamus, where it is co-localized with the Y1 receptor. The Y5 receptor mediates the effects of NPY-induced food intake, with selective agonism of this receptor, resulting in stimulation of feeding (105). However, full-length PYY_1–36_ does not appear to influence appetite when administered peripherally, suggesting it is unable to access these orexigenic receptors (106).

The most notable role of PYY_3–36_ is as an anorectic peptide. Acute peripheral administration of PYY_3–36_ to rodents or humans reduces food intake (46, 107). Intermittent exogenous administration of PYY_3–36_ reduces food intake, body weight, and adiposity in rats, and prevents weight gain in diet-induced obese rats (108, 109). However, the primary mechanism by which PYY_3–36_ reduces food intake is unclear. It appears to be mediated by Y2 receptors; the anorectic effect of PYY is absent in mice with targeted gene deletion of the Y2R (46, 107). Initial studies found that PYY increased POMC mRNA and induced electrophysiological activation of these neurons. It was suggested that the anorectic effects were due to activation of pre-synaptic inhibitory Y2 receptors on arcuate NPY/AgRP neurons, resulting is a reduced inhibition of POMC neurons by the inhibitory neurotransmitter, γ-aminobutyric acid (GABA) (107). However, subsequent studies revealed that mice lacking functional POMC or MC4R were as susceptible as wild-type mice to the acute anorectic effects of peripheral PYY_3–36_ administration (110, 111). This indicates that although PYY_3–36_ may stimulate melanocortin production, melanocortin signaling is not necessary for its anorectic effects.

It has also been suggested that PYY_3–36_ acts to reduce food intake by signaling via peripheral neurons. The Y2R has been located on vagal afferent fibers, however, conflicting evidence has been found as to whether PYY_3–36_ requires an intact vagus to signal a reduction in food intake. Total subdiaphragmatic vagotomy did not attenuate the reduction in food intake compared to sham operated mice, and instead was found to prolong the anorectic effects of PYY_3–36_. Hence, vagal tone may modulate the duration of action of intestinally secreted PYY_3–36_, but may not be required for short-term signaling (59). However, in a separate study, bilateral subdiaphragmatic vagotomy abolished both the anorectic effects of PYY_3–36_ and its ability to induce Fos expression in the ARC of rats following peripheral administration, indicating blockade of hypothalamic activation (112). Interestingly, a novel mechanism by which PYY-releasing cells may directly influence neuronal function has recently been proposed. PYY-expressing EECs were found to exhibit long cytoplasmic processes with some characteristics of neuronal processes, named neuropods, which directly contact enteric neurons (Figure 3). Culturing PYY-expressing EECs isolated by FACS with sensory neurons resulted in the development of neuropods, which contacted the neuronal neurites, from where a putative axon develops. These EEC–neuron connections suggest that L-cells may have the ability to directly participate in the transmission of sensory signals from the gut lumen (6, 7).

It has been proposed that PYY_3–36_ induces hypophagia by causing non-specific malaise. Conditioned taste aversion (CTA) protocols are commonly used as a paradigm for nausea in rodents, which lack the necessary anatomy for vomiting. Infusions of PYY_3–36_ have been dose-dependently associated with CTA in rats and mice, thought in part to be due to inhibition of gastric emptying (59, 113). A similar dose-dependent effect on nausea and abdominal discomfort has been found following exogenous administration of PYY_3–36_ in humans (49). The aversive effects of this peptide potentially limit its therapeutic use as an anti-obesity agent. However, interestingly, there is little connection between the nausea experienced and the level of food intake inhibition, suggesting that perhaps the nausea occurs at a threshold level, but is unconnected to the physiological satiating effects of PYY_3–36_ (13).

## Nutrient-Sensing by L-Cells

Currently, the most effective treatment for obesity is bariatric surgery; the Roux-en Y-Gastric bypass is the most commonly performed procedure, and results in sustained weight loss, though the popularity of the vertical sleeve gastrectomy is increasing (114). In healthy individuals, the post-prandial response involves a complex cocktail of hormones, their release reflecting the ingested and absorbed macronutrients (115). The post-prandial GLP-1 and PYY response is reported to be blunted in obese patients (116). However, following bariatric surgery, patients exhibit increased post-prandial levels of anorectic gut hormones GLP-1 and PYY, and attenuated levels of ghrelin, an orexigenic hormone released from the stomach (117). In addition, amelioration of type 2 diabetes frequently occurs within days of surgery. It has been widely postulated that altered post-prandial gut hormone levels may be responsible for at least some of the metabolic effects of bypass surgery. EECs are therefore a key area of interest in research into alternatives to bariatric surgery. Changes in intestinal morphology, in particular villus hyperplasia, have been implicated in the adaptive response following Roux-en Y-Gastric bypass and ileal interposition in rats (118, 119). A shift from absorptive to more secretory cell lineages, such as goblet cells, has also been observed (119, 120). In rats, villus proliferation occurs following the implantation of a duodenal–endoluminal sleeve, a device that acts as a physical barrier between nutrients and absorptive tissue (121). Increased villus length and surface area in these studies are associated with beneficial metabolic changes, including improved glucose homeostasis and increased post-prandial GLP-1 secretion (122). Exploiting the mechanisms by which various dietary macronutrients activate GPCRs on L-cells, and hence, the release of endogenous GLP-1 and PYY represents a possible therapeutic target. It is thus important to understand the specific mechanisms by which L-cells sense different types of nutrients (23).

Receptors on the apical surface of open-type L-cells directly sense dietary components in the intestinal lumen, and respond to produce the appropriate endocrine response (Figure 1). The contents of the intestinal lumen vary considerably with diet, requiring a number of specific receptors to detect the different macronutrients that modulate the secretion of hormones. Secretion of GLP-1 and PYY by L-cells is preceded by raised intracellular calcium and cyclic adenosine monophosphate (cAMP) levels. Calcium is released from intracellular stores following membrane depolarization due to increased sodium-dependent cell excitability and calcium influx, leading to the exocytosis of hormone-containing vesicles. This signal is potentiated downstream by intracellular cAMP, which is increased by the action of G_s_ protein coupled receptors, further augmenting hormone release (20, 123). Recently, super-resolution microscopy has demonstrated that secretory vesicles from L-cells of the mouse, rat, pig, and human contain primarily either GLP-1 or PYY. This raises the possibility that either hormone may be selectively released from a microdomain of a single EEC (124). A specific combination of cellular machinery may need to be activated in the L-cell to cause the differential release of GLP-1 or PYY. However, how these signals are integrated remains to be elucidated.

## Protein-Sensing Receptors

Increasing dietary protein content by 10–15% promotes satiety, reduces overall calorie intake, and produces sustained weight loss in rodents and man, possibly due in part to the induced changes in circulating gut hormones (125–127). In both healthy and obese subjects, a high-protein meal increases plasma PYY levels significantly more than an isocaloric meal high in carbohydrate or fat, while PYY null mice are resistant to the satiating effects of protein (128). Chronic exposure to a high-protein diet also elevates post-prandial GLP-1 and increases satiety levels in healthy subjects (129).

### The peptone receptor

It has been shown that peptones stimulate the release of CCK from I-cells, and stimulate GLP-1 release from *ex vivo* rat small intestine and colon, and from STC-1 cells. Activity of the proglucagon gene promoter is also enhanced, leading to increased transcription (130). The peptone GPCR, GPR93, is highly expressed by cells of the duodenal mucosa, including L-cells. Activation of GPR93 by protein hydrolyzates, results in the transcription and release of CCK (24, 131). Peptide-transporter 1 (PEPT1) is a brush-border transporter of di- and tri-peptides, located on L-cells of the small intestine and colon. Primary murine L-cell cultures have been shown to release GLP-1 in response to peptone administration, via PEPT1-dependent electrogenic uptake and activation of the CaSR (132). Hence, the larger fragments of protein hydrolysis may directly regulate proglucagon synthesis in the gut, and influence the secretion of its posttranslational products (130).

### The mu-opioid receptor

The oligopeptides produced by protein breakdown are also active at the mu-opioid receptor (MOR), an inhibitory GPCR. The MOR is present in the small intestine and brain, particularly the nucleus accumbens. Agonism and antagonism of the central MOR has been shown to increase and decrease food intake, respectively (133, 134). Duraffourd et al. demonstrated that MORs located in the walls of the portal vein respond to the products of protein digestion *in vivo*, to induce intestinal gluconeogenesis (135). This occurs as a result of a protein-enriched diet, and leads to detection of increased portal glucose and signaling to the hypothalamic nuclei, which regulate food intake (136). These effects are abolished following denervation of the portal vein, providing a plausible link between the assimilation of dietary protein in the gut, and the central induction of satiation (136).

### The CaSR

The CaSR seems to promote the secretion of GLP-1 and PYY, and has thus been identified as a potential therapeutic target in the treatment of diabetes and obesity (23). The CaSR is proposed to act as an l-amino acid sensor in the gut, and has been identified on rodent and human L-cells (Figure 1). The CaSR is able to bind a wide range of amino acids. However, the most potent are the aromatic amino acids, l-phenylalanine and l-tryptophan (26). Mace et al. reported that CaSR activation by the l-amino acids phenylalanine, tryptophan, asparagine, arginine, and glutamine, resulted in the secretion of the GLP-1 and PYY. These responses were abolished in the presence of a CaSR inhibitor or the absence of extracellular calcium, identifying amino acids as allosteric agonists that require the additional presence of calcium to initiate their effects (137). Voltage clamping of intact murine mucosa has shown that the response of the CaSR to l-glutamine is glucose sensitive. Peptone-triggered secretion of GLP-1 from murine primary colonic cultures is also abolished in the presence of CaSR antagonists and removal of extracellular calcium, though whether this effect represents the sensing of individual amino acids generated by initial protein breakdown, or the ability of small peptides to bind to the CaSR is unclear (132).

### The GPRC6a

G-protein coupled receptor family C group 6 subtype A (GPRC6a) is closely related to the CaSR, though it preferentially binds the basic amino acids, l-arginine, l-lysine, and l-ornithine, whereas aromatic amino acids are inactive at this receptor (25). Similarly to the CaSR, GPRC6a possesses a calcium binding site on its extracellular domain, although with a weaker affinity than the aforementioned receptor, suggesting overlapping functions (138). GPRC6a is widely expressed throughout the body, including the gut, liver, spleen, lung, heart, kidney, skeletal muscle, brain, and bone (138–140). Exon II of *GPRC6a* encodes part of an orthosteric binding site for endogenous ligands. Deletion of exon II in GPRC6a knockout mice triggers the development of a complex metabolic-like syndrome. This affects multiple organs, and results in consequences including demineralization of bone, hyperglycemia, and obesity, suggesting that GPRC6a plays a role in the coordination of nutrient sensing and metabolism in these tissues (141). However, an additional GPRC6a knockout, involving disruption of the 7-transmembrane and C-terminal region by deletion of exon VI, displays a normal bone phenotype and glucose tolerance (142). The expression of GPRC6a in the gut, and its activation by l-amino acids indicated that this receptor may mediate the effects of fluctuating dietary protein on energy homeostasis. The GPRC6a, closely related to the CaSR, has been cloned from human and rodent cells, and subsequently localized on the GLUTag L-cell line (139). Application of l-ornithine to a homologous model of the human GPRC6a leads to coupling of the receptor to G_q_. The consequent activation of phospholipase C and increased intracellular calcium then stimulates the secretion of GLP-1 (25, 26). Pharmacological inhibition of the downstream pathway decreases the resulting GLP-1 exocytosis from GLUTag cells. Furthermore, following administration of l-ornithine and l-lysine, depletion of endogenous GPRC6a by a small interfering RNA attenuated the intracellular calcium response (143). However, studies using mice with the entire GPRC6a gene deleted, found that this receptor is not necessary for the effects of a high- or low-protein diet on body weight (144).

### The taste receptors

The capacity to sense the composition of food via taste allows for the selection of essential nutrients in the diet, in addition to the avoidance of harmful substances. Family C of the GPCRs also encompasses the type 1 taste receptors (T1Rs), formed of the subunits T1R1, T1R2, and T1R3. The combination of subunits T1R1 with T1R3 forms the umami taste receptor, a known l-amino acid receptor expressed in the lingual epithelium (26, 145). The umami receptor is broadly responsive to aliphatic amino acids, but is particularly sensitive to l-glutamine and l-aspartate, the taste of which gives the umami receptor its name and enhances food palatability (146). The rodent and human T1Rs are only approximately 70% homologous, resulting in varying agonist affinities (147). The human T1R1 is considerably more sensitive to glutamate than other amino acids, while the T1R2 is sensitive to artificial sweeteners, such as aspartame and cyclamate (148). Gustducin is a G-protein that plays a role in the downstream signaling transduction of taste from T1Rs. Coupling of the T1Rs to gustducin is thought to stimulate phosphodiesterase activity, leading to the hydrolysis of cAMP (149). Coexpression of T1R1/T1R3 with gustducin occurs in the fungiform and palatal taste buds and has also been reported in the GLP-1-producing STC1 cell line (150–152). However, it is currently unclear whether T1R1/T1R3 plays a physiological role in L-cell sensing of amino acids.

### Specific receptors

In addition to promiscuous amino acid receptors, specific amino acid sensors are expressed in the gut. Glutamate is the primary excitatory neurotransmitter in the central nervous system; however, its receptors are also widely expressed in the periphery, including the GI tract. Metabotropic glutamate receptors are GPCRs that are classified into three groups. Group III includes the metabotropic glutamate receptor 4, mGluR4, which is highly expressed in the distal gut, with highest expression in the proximal colon of mice and humans (153). Activation of mGluR4 by glutamate decreases intracellular cAMP production, through coupling to G proteins, which inhibit adenylyl cyclase activity (154). Glutamate is found in many food sources, including legumes and dairy products, and is converted to glutamine in the gut, liver, and kidneys (155, 156).

As a major product of protein digestion, glutamine is an important fuel source for the gut that can enhance protein synthesis, particularly after injury (157). Glutamine acts as a GLP-1 secretagogue in primary cell cultures and the GLUTag cell line, causing both initiation and further amplification of GLP-1 secretion, even at physiologically relevant levels (20, 123). The mechanism is unclear, though the initial step involves electrogenic uptake of the amino acid, followed by a downstream enhancing step, which in primary murine L-cells involves an increase in cytosolic Ca^2+^ and cAMP (20, 123). In further human studies, oral glutamine was found to be well tolerated, and to result in a biphasic increase of GLP-1 in the circulation of lean, obese, and diabetic subjects (27). Glutamine is commonly delivered as part of enteral and parenteral nutrition, due to its ability to preserve the integrity of the gut, which may be attributable to the co-release of GLP-2 with GLP-1 (27, 158).

l-arginine is defined as a conditionally essential amino acid, as the bodies’ ability to synthesize it varies with age and injury status (159, 160). l-arginine is involved in the synthesis of nitric oxide, as well as that of several other amino acids, including l-glutamate, l-ornithine, proline, and creatine (161). A powerful secretagogue, it has long been known that l-arginine promotes the secretion of insulin and glucagon from the β- and α-cells of the pancreas, respectively, which may be partly due to its potent action at the GPRC6a receptor (162–164). Oral l-arginine stimulates the secretion of GLP-1 and insulin in lean- and diet-induced obese mice *in vivo*. This effect was abolished in *Glp1r* knockout mice, and hence, the improvement of l-arginine-stimulated glucose tolerance is dependent on this receptor (22). Furthermore, the addition of l-arginine to a low-protein diet was associated with a reduction in white adipose tissue and increased energy expenditure (165). Dietary supplementation of amino acids, such as glutamine and l-arginine, which potentiate the release of GLP-1 and PYY *in vivo*, may be useful as a nutritional therapy to enhance the response of the gut endocrine response in obese patients.

### Mammalian target of rapamycin

Regulation of protein synthesis requires cells to sense nutrient availability. The mammalian target of rapamycin (mTOR) is a serine/threonine kinase that regulates cellular capacity for protein biosynthesis and cell growth by sensing intracellular amino acid levels. Autophagy involves the degradation of cellular protein into amino acids for use during conditions of starvation, a process inhibited by mTOR in an amino acid-rich environment. The levels of branched-chain amino acids, such as leucine, fall the most rapidly, with their depletion being the first to cause activation of mTOR (166). Dietary supplementation of branched-chain amino acids following exercise has anabolic effects on human muscle, involving phosphorylation of mTOR (167). The anticancer drug rapamycin mimics the conditions of low-amino acid availability or limited nitrogen, restricting cell growth and inducing autophagy (168). l-glutamine is the preferred nitrogen source in cells, the balance of which provides the rate-limiting step for anabolic conditions (168). Uptake, and subsequent efflux, of l-glutamine is required for the activation of mTOR by essential amino acids, leading to tissue growth (21).

### Adenosine monophosphate-activated protein kinase

Further regulation of protein metabolism occurs via adenosine monophosphate-activated protein kinase (AMPK), a sensor of adenosine nucleotides. Hydrolysis of adenosine triphosphate (ATP) increases the intracellular ratio of AMP to ATP, leading to the activation of AMPK (19). This negatively regulates downstream targets of TOR complex 1 (TORC1), and thus, responds to energy deficits by suppressing cell growth (169, 170). These signaling pathways converge further, through the regulation of Forkhead box class O (FoxO) transcription factors. Activation of AMPK and TORC1 leads to the phosphorylation of FoxO and the elevated expression of its transcription factors, respectively, with both processes acting to inhibit cell proliferation (171, 172). Thus, AMPK acts as a nutritional sensor that influences protein metabolism and may interact with protein-sensing mechanisms.

A thorough understanding of the ability of the cell surface and intracellular sensors, which detect the products of protein digest to regulate energy balance by responding to nutritional and hormonal signals may lead to new ways of exploiting the benefits of high-protein diets without requiring patients to adopt such regimes. Simultaneous pharmacological targeting of receptors enriched in the gut and pancreas, which have dual functions in the release of anorectic peptides from EECs and incretin effects, might be beneficial in diabetic obese patients.

## Carbohydrate-Sensing Receptors

Most mammalian cells rely on a steady glucose supply as an energy source, though circulating levels must be kept low in order to avoid the toxic state of hyperglycemia (173). Carbohydrate sensing is directly related to glucose homeostasis, beginning with taste receptors in the mouth and subsequently glucose sensors in the gut. Activation of sweet taste receptors stimulates incretin hormone release from EECs and promotes glucose absorption via increased intestinal expression of the transporters, GLUT2 and SGLT1 (119, 174). The tight, short-term control of glucose homeostasis allows sufficient flux of glucose to the brain, while avoiding states of hyperglycemia. However, there is no clear link between carbohydrate sensing and longer-term appetite regulation. An increase in blood glucose following either the consumption of carbohydrate in the form of breakfast cereal or intravenous infusion of glucose is not associated with decreased food intake, indicating that the glycemic response is not directly related to satiety (175, 176). Obese patients who have undergone Roux-en-Y gastric bypass are commonly reported to have resolution of the associated type 2 diabetes within days of the procedure. This precludes any weight loss, and may be partly due to the effects of duodenal isolation on acute intestinal glucose sensing and transport, resulting in the improved regulation of glycemia (119, 177).

### Sodium-glucose transporter 1

Integrated glucose homeostasis relies on the ability of multiple tissue types to sense glucose levels. However, before glucose disposal can occur, dietary carbohydrate must first be metabolized and absorbed from the gut lumen. Hydrolysis of carbohydrates by small intestinal brush-border enzymes produces monosaccharides, such as glucose, that can then be absorbed. An essential component of this transepithelial transport system is the co-transporter, SGLT-1, which uses the sodium electrochemical gradient produced by the Na^+^/K^+^ ATPase pump to allow glucose entry across the apical membrane and into enterocytes (178, 179). Facilitated glucose transporters located on the basolateral membrane, such as GLUT2, subsequently allow passive diffusion of glucose into the interstitial space (180).

Enteroendocrine L-cells are directly glucose-responsive. Glucose-sensing leads to membrane depolarization and calcium entry through voltage-gated channels, leading to exocytosis of GLP-1-containing vesicles (27). Moriya et al. determined that *in vivo* injection of glucose into the upper intestine of mice increased circulating GLP-1 and GIP levels, and that this effect was prevented by coadministration of an SGLT-1 inhibitor. Injection of glucose into the colon did not affect incretin levels, consistent with localization of SGLT-1 mRNA in L-cells of the small intestine and very low detection in the colon (181). The non-metabolizable glucose analog and SGLT-1 agonist, α-methylglucopyranoside, is also a potent stimulator of GLP-1 release from primary intestinal L-cell cultures, highlighting this transporter as a major stimulator of hormone release from EECs (27).

### ATP-sensitive potassium

The ATP-sensitive potassium (K_ATP_) channel subunits, Kir6.2 and Sur1, have been identified in primary L-cells expressed at levels similar to those seen in pancreatic cells, where they are known to be involved in insulin release (182). Tolbutamide, which blocks these channels, triggered GLP-1 release from primary L-cells, confirming the presence of functional K_ATP_ channels in L-cells. However, the exact function of these channels is unknown (27).

### The sweet taste receptor

Heterodimerization of subunits T1R2 and T1R3 of the abovementioned family C of the GPCRs, forms the sweet taste receptor. The sweet taste receptor is expressed on the lingual epithelium, but also acts as a carbohydrate sensor in the gut and STC1 cell line (151). Coupling of T1R2/T1R3 to the taste-associated G-protein, gustducin, mediates second messenger signaling cascades, and allows for the secretion of GLP-1 in the presence of glucose. This response is defective in *gustducin*-null mice, which have an impaired GLP-1 response to luminal glucose (152). Reimann et al. reported that although L-cells were unresponsive to artificial sweeteners at low concentrations, higher concentrations resulted in GLP-1 secretion *in vitro*, an effect that was additive with glucose, and may be due to activation of the sweet taste receptor (27). However, studies by Fujita et al. indicated that oral administration of a range of sweeteners active at sweet taste receptors on the lingual epithelium, did not result in incretin secretion *in vivo* in the rat (183). The role of carbohydrate sensing in the gut on the long-term regulation of energy homeostasis is thus currently unclear.

## Fatty Acid-Sensing Receptors

### Ethanolamide receptors

The varying chain length and saturation of fatty acids and their derivatives confer distinct receptor affinities. The G_s_-associated GPCR, GPR119, detects the endogenous saturated fatty-acid ethanolamides, such as oleoylethanolamide (OEA) (Figure 1). OEA is produced in the small intestine, and has a chain length of 18 carbons, with one double bond. This high degree of saturation produces the greater efficacy of OEA at GPR119 than other ethanolamides (184). GPR119 is present on both pancreatic β-cells and intestinal L-cells. Activation in the pancreas mediates insulin secretion in the presence of glucose, via raised intracellular cAMP (185). Activation of GPR119 in the gut by the products of fat hydrolysis, leads to the release of GLP-1 and insulin secretion in a glucose-dependent manner (186). Conversely, GPR119 agonists are able to stimulate GLP-1 secretion in the absence of nutrients in GLUTag cells (187). Daily intraperitoneal administration of OEA to mice induces satiety and prevents weight gain, a feature that may be true of other GPR119 agonists (184, 188). Stimulation of GPR119 is coupled to increased proglucagon expression in GLUTag cells (189). This is further supported by the attenuated GLP-1 secretion in GPR119 knock-out mice in response to glucose. GPR119 may hence control GLP-1 synthesis, and pharmacological activation of this receptor may therefore enhance glycemic control and reduce food intake in diabetic patients (190).

### SCFA receptors

Short-chain fatty acids (SCFAs) comprise a chain of fewer than six carbons in length. They are produced by the bacterial fermentation of undigested carbohydrates, the main products being acetate, propionate, and butyrate (191). Putative receptors for SCFAs, include the free-fatty acid receptors (FFARs) 2 and 3, also known as GPR43 and 41, respectively. Both receptors couple to G_i/o_, and activation thus leads to raised intracellular calcium and decreased cAMP. FFAR2 also exhibits dual coupling to G_q_, an activator of phospholipase C (192). Localized in the human colon and ileum, FFAR2 and 3 are expressed by enteroendocrine L-cells that secrete PYY. However, FFAR3 is much more enriched in L-cells of the small intestine (29), while FFAR2 is expressed at higher levels in the human colon. Coexpression of FFAR2 and 3 has not been observed in the same cells (191, 193). Recent evidence has suggested that increased intake of dietary fiber may reduce appetite by increasing the SCFAs produced by microbial fermentation in the colon, and thus, increasing the secretion of anorectic gut hormones (194). Propionate, in particular, may play an important role in satiety. Colonic infusion of SCFAs increases circulating PYY levels *in vivo* in the rat, inhibiting upper GI tract motility consistent with the ileal brake (28). Acetate and propionate enhance GLP-1 secretion in murine primary cell cultures. Propionate stimulates the secretion of GLP-1 and PYY from rodent colon *in vivo* and *in vitro*. This effect is significantly reduced in *Ffar2*-null mice, suggesting FFAR2 is important in SCFA-induced gut hormone secretion (30). The stimulatory effects of acetate on basal and glucose-stimulated GLP-1 release are also abolished in primary L-cell cultures from FFAR2 knock-out mice, though they were also found to be significantly reduced in FFAR3 knock-out tissue (29). Chronic delivery of propionate to the proximal colon of obese human subjects increases the production of PYY and GLP-1, reduces energy intake, and prevents weight gain. Targeted colonic propionate may therefore represent a novel and effective approach for weight management (195).

### MCFA and LCFA receptors

Medium chain fatty acids (MCFAs) and long-chain fatty acids (LCFAs) are agonists of the FFAR1 and 4, previously known as GPR40 and 120. An MCFA is formed of a 6–12 carbon chain, and LCFAs of a chain containing more than 12 carbons (196). FFAR1 is expressed in the pancreatic β-cell, where evidence suggests it can influence glucose-stimulated insulin release (197). The effects of hyperlipidemia on glucose homeostasis may in part be mediated by this receptor (198, 199). FFAR1 is also expressed in GLP-1- and GIP-expressing cells of the GI tract, and the secretion of these hormones in response to oral fat is attenuated in *Ffar1*-null mice. The activation of FFAR1 in EECs therefore provides a route for the indirect regulation of insulin secretion (32). FFAR4 is expressed in the δ-cells of the pancreas, and widely in EECs. FFAR4 responds to unsaturated MCFAs and LCFAs by coupling to Ga_q_ and increasing intracellular calcium, leading to GLP-1 secretion (31, 33, 37). FFAR4-deficient mice gain more weight than wild-type controls, and show higher fasting plasma levels of glucose and insulin when fed a high-fat diet (200). Human obesity has been associated with altered FFAR4 expression in adipose tissue, involving a deleterious *Ffar4* mutation that is unable to transduce LCFA binding (200). While it feels counter intuitive to use lipids to treat obesity and metabolic disease, exploiting the pathways by which dietary lipid is sensed and drives satiety may be useful in the development of new pharmaceutical or nutraceutical agents.

## Summary

Currently, the most effective treatment for obesity is bariatric surgery, specifically the Roux-en Y Gastric bypass. However, this method is not suitable for all, due the highly invasive nature and associated risks for patients, particularly those with cardiovascular problems. Due to the rapid degradation of endogenous PYY and GLP-1, and the difficulties in administration of peptide-based drugs, exploiting the mechanisms that lead to their continued release, such as the GLP-1 receptor agonist, Saxenda, might be a useful alternative approach to treating patients suffering from obesity and impaired glucose tolerance. Combination of pharmacological treatments that act by targeting the nutrient-sensing receptors and transporters discussed above may ameliorate the defective satiety and glucose homeostasis pathways present in these patient groups. Likewise, the potential formulation of nutraceuticals, containing specific combinations of amino acids and other nutrient receptor ligands, may represent another approach (Table 1). The continued receptor deorphanization and elucidation of the cellular machinery by which the L-cell detects nutrients may thus prove valuable in the development of new treatments for diabetes and obesity.

## Conflict of Interest Statement

The authors declare that the research was conducted in the absence of any commercial or financial relationships that could be construed as a potential conflict of interest.

## Abbreviations

AgRP, agouti-related peptide; AMPK, adenosine monophosphate-activated protein kinase; AP, area postrema; ARC, arcuate nucleus; ATP, adenosine triphosphate; cAMP, cyclic adenosine monophosphate; CCK, cholecystokinin; CTA, conditioned taste aversion; DPPIV, dipeptidyl peptidase IV; EEC, enteroendocrine cell; FACS, fluorescence-activated cell sorted analysis; FFARs, free fatty-acid receptors; FoxO, forkhead box class O; GABA, γ-aminobutyric acid; GI, gastrointestinal; GIP, gastric inhibitory peptide; GLP-1, glucagon-like peptide 1; GLP-2, glucagon-like peptide 2; GLUT2, glucose transporter 2; GPCR, G-protein coupled receptors; K_ATP_, ATP-sensitive potassium channel; LCFAs, long-chain fatty acids; MCFAs, medium chain fatty acids; MOR, mu-opioid receptor; mTOR, mammalian target of rapamycin; NPY, neuropeptide Y; NTS, nucleus of the solitary tract; OEA, oleoylethanolamide; PEPT1, peptide-transporter 1; POMC, pro-opio melanocortin; PYY, peptide YY; SCFAs, short-chain fatty acids; SGLT-1, sodium-glucose transporter 1; T1Rs, type 1 taste receptors; TORC1, TOR complex 1.
